# Supplementary material for: NcPath: a novel platform for visualization and enrichment analysis of human non-coding RNA and KEGG signaling pathways
Source: Bioinformatics. 2022 Dec 16;39(1):btac812. doi: 10.1093/bioinformatics/btac812 (PMC9825761; doi:10.1093/bioinformatics/btac812)
Supplement: btac812_Supplementary_Data [file btac812_supplementary_data.docx]

**Screening of the reliable relationship between lncRNA and mRNA**

**The distribution of** **lncRNA-mRNA interaction scores：**

10,000 scores of lncRNA-mRNA interaction were randomly selected from the five interaction categories of Co-expression, Co-HM, CO-TF and LncPro score and ceRNA (p-value of hypergeometric test) respectively, and their probability density distribution plots were shown as follows:


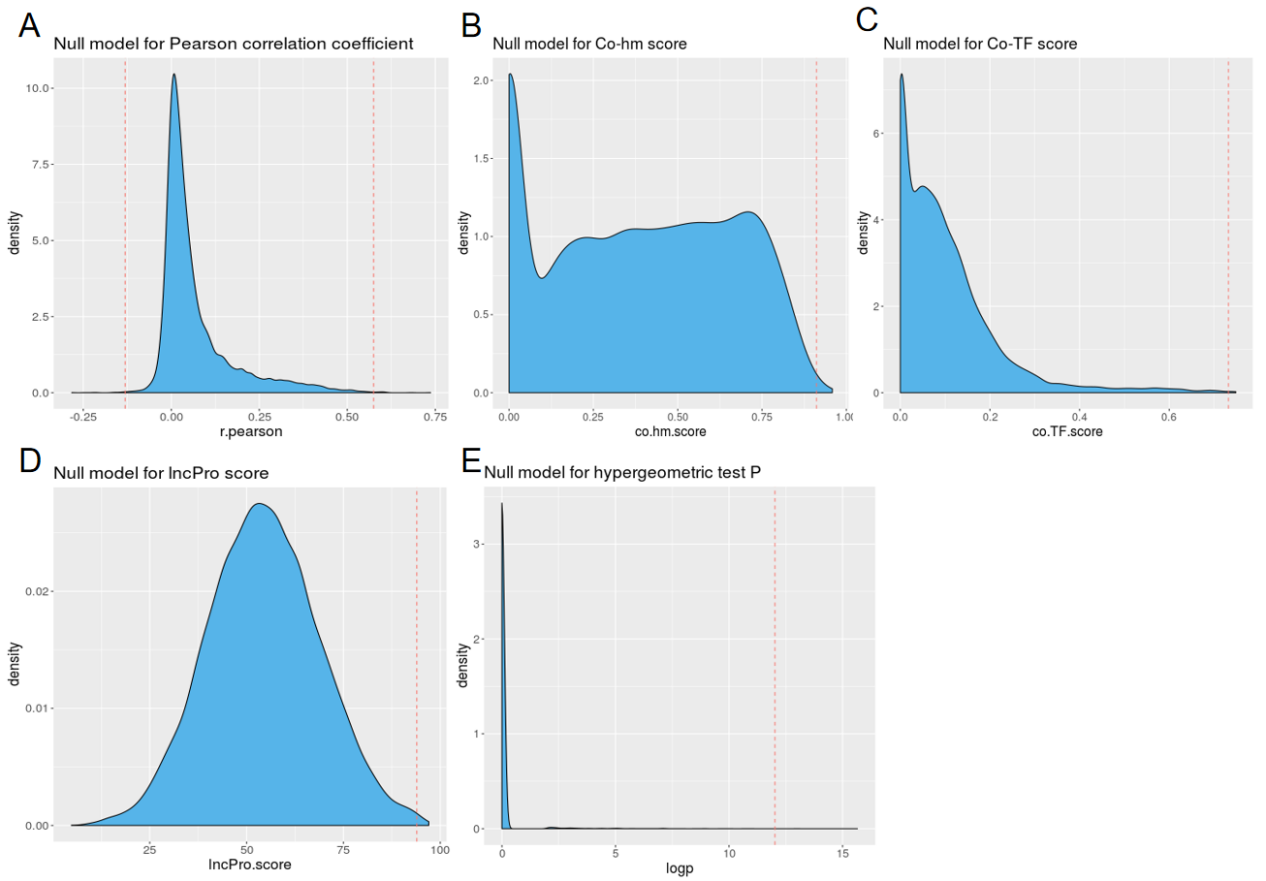


**Figure S1. The probability density distribution plots of five interaction categories.** (A-E) The probability density distribution plots of Co-expression, Co-HM score, Co-TF score, LncPro score and ceRNA.

**Reliable lncRNA-mRNA interaction pairs:**
For the above five kinds of interaction relations, Monte Carlo simulation was used to evaluate whether a lncRNA-mRNA pair was significantly reliable. We chose 10, 000 scores randomly each time to the cutoff score of P-value=0.001. This process was repeated 10, 000 times and the average cutoff value is finally determined (see the red line in the figures above, and the table below). Bilateral test was used for co-expression, and the other 4 lncRNA-mRNA interaction relations were tested on the right side.

**Table S1. The average cutoff values of five interaction relations.**

| **Interaction relations** | TF | Hm | Cor | Lncpro | ceRNA |
| --- | --- | --- | --- | --- | --- |
| **Cutoff** | 0.7322795 | 0.9114914 | 0.5748191  -0.1311137 | 93.93853 | 11.9 |

With the above cutoff as the boundary, the lncRNA-mRNA pairs with significant scores in the five interaction relationships were considered to be reliable, that is, the authentic relationship pairs of lncRNA-mRNA interaction in each interaction category. We finally obtained seven types of reliable interactions, including the five relationships mentioned above, cis-regulation interactions and clip-validated interactions. Then the overlap between the seven types of reliable lncRNA-PCG interactions can be seen in the following figure.


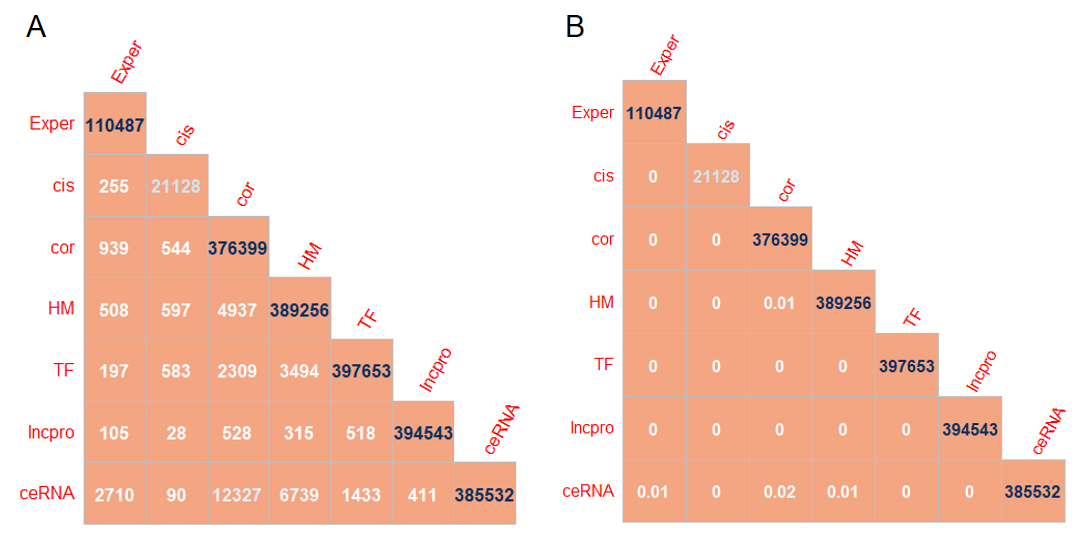


**Figure S2. Then the overlap between the seven types of reliable lncRNA-PCG interactions.** (A)The overlap number between reliable lncRNA-PCG interactions derived from 7 methods. (B)The similarity between reliable lncRNA-PCG interactions derived from 7 methods.

The dark blue number on the main diagonal represents the number of reliable lncRNA-PCG interaction pairs obtained by each method. The similarity in Figure B is defined as:

$$\text{similarity(S},\text{ T) =}\frac{\left|\text{S}\cap\text{T}\right|}{\left|\text{S}\cup\text{T}\right|}$$

S and T represent the lncRNA-PCG interaction pair sets obtained by two different methods respectively.

The above results showed that the reliable lncRNA-PCG interaction pairs obtained by different methods had almost no overlap (the highest similarity between the ceRNA and co-expression was 2%), that is, the reliable lncRNA-PCG interaction obtained from 7 different methods had a relatively large difference.

A total of 1985342 reliable lncRNA-PCG interaction pairs from 7 methods were identified (among them, we used 556798 LTIs related to the PCGs in 222 pathways). Then, we observed the number distribution of PCGs interacting with each lncRNA using the reliable interaction pairs (see **Figure S3**). Most lncRNAs had reliable interaction with a small number of PCGs, and a few lncRNAs had reliable interaction with a large number of PCGs. There were 2722 lncRNAs that had no reliable interaction with any PCGs (this part of lncRNAs can be considered as having weak regulatory function), accounting for 15% of the total number of lncRNAs. There are 2879 lncRNAs with more than 100 reliable interaction mRNAs, accounting for 16% of the total lncRNAs, which may have strong regulatory function.


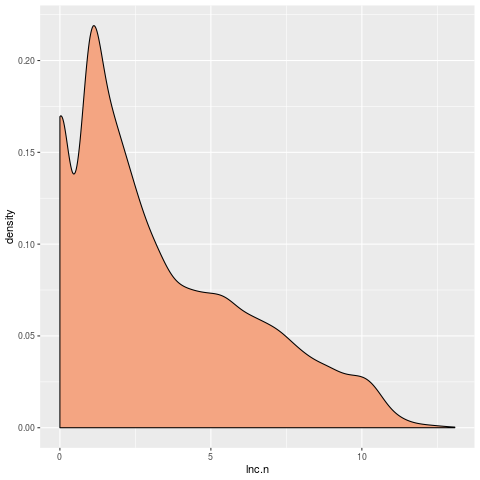


**Figure S3.** **The number distribution of PCGs interacting with each lncRNA using the reliable interaction pairs.**

**Table S2. The top pathways derived from mRNA alone.**

|  | p-value | pathway |
| --- | --- | --- |
| 1 | 1.121e-05 | Protein digestion and absorption |
| 2 | 2.321e-05 | Amoebiasis |
| 3 | 2.375e-04 | Focal adhesion |
| 4 | 0.000532 | AGE-RAGE signaling pathway in diabetic complications |
| 5 | 0.000432 | Small cell lung cancer |
| 6 | 0.001082 | Relaxin signaling pathway |
| 7 | 0.001452 | ECM-receptor interaction |
| 8 | 0.001891 | Human papillomavirus infection |
| 9 | 0.002344 | PI3K-Akt signaling pathway |
| 10 | 0.010164 | Proteoglycans in cancer |
| 11 | 0.013984 | GnRH secretion |
| 12 | 0.013402 | Bacterial invasion of epithelial cells |
| 13 | 0.033220 | Phagosome |
| 14 | 0.038379 | Diabetic cardiomyopathy |
| 15 | 0.042205 | MicroRNAs in cancer |

Since only 15 pathways were enriched through the mRNA list, we present all enriched pathways in Table S2.

**Table S3. The top-30 pathways derived from miRNA alone.**

|  | p-value | pathway |
| --- | --- | --- |
| 1 | 1.3054e-16 | Cell cycle |
| 2 | 5.1173e-12 | Pancreatic cancer |
| 3 | 8.9762e-11 | Prostate cancer |
| 4 | 5.4534e-10 | Chronic myeloid leukemia |
| 5 | 5.166e-10 | Cellular senescence |
| 6 | 1.7616e-08 | Hepatocellular carcinoma |
| 7 | 3.3873e-08 | Focal adhesion |
| 8 | 3.5878e-08 | AGE-RAGE signaling pathway in diabetic complications |
| 9 | 5.1520e-08 | Endocrine resistance |
| 10 | 1.3554e-07 | Hippo signaling pathway |
| 11 | 1.2325e-07 | Gastric cancer |
| 12 | 2.3620e-07 | Small cell lung cancer |
| 13 | 2.8287e-07 | Adherens junction |
| 14 | 3.6676e-07 | PI3K-Akt signaling pathway |
| 15 | 4.4875e-07 | FoxO signaling pathway |
| 16 | 7.7162e-07 | Human papillomavirus infection |
| 17 | 1.0126e-06 | p53 signaling pathway |
| 18 | 1.5296e-06 | EGFR tyrosine kinase inhibitor resistance |
| 19 | 1.4319e-06 | Prolactin signaling pathway |
| 20 | 1.5585e-06 | Regulation of actin cytoskeleton |
| 21 | 1.3855e-06 | Colorectal cancer |
| 22 | 1.6573e-06 | Kaposi sarcoma-associated herpesvirus infection |
| 23 | 3.2945e-06 | HIF-1 signaling pathway |
| 24 | 3.2455e-06 | Glioma |
| 25 | 4.3445e-06 | MAPK signaling pathway |
| 26 | 1.5541e-05 | Thyroid hormone signaling pathway |
| 27 | 1.5646e-05 | Ras signaling pathway |
| 28 | 1.4595e-05 | Acute myeloid leukemia |
| 29 | 2.3264e-05 | Bladder cancer |
| 30 | 4.5160e-05 | Endometrial cancer |

**Table S4. The top-30 pathways derived from lncRNA alone.**

|  | p-value | pathway |
| --- | --- | --- |
| 1 | 1.0452e-09 | AMPK signaling pathway |
| 2 | 1.1876e-09 | mTOR signaling pathway |
| 3 | 2.3198e-08 | Adherens junction |
| 4 | 3.7974e-08 | Longevity regulating pathway |
| 5 | 2.1838e-07 | Chronic myeloid leukemia |
| 6 | 4.2283e-07 | Sphingolipid signaling pathway |
| 7 | 4.4511e-07 | Neurotrophin signaling pathway |
| 8 | 4.4911e-07 | Insulin resistance |
| 9 | 7.0055e-07 | Hippo signaling pathway |
| 10 | 9.3600e-07 | Thyroid hormone signaling pathway |
| 11 | 9.7223e-07 | EGFR tyrosine kinase inhibitor resistance |
| 12 | 1.0674e-06 | Kaposi sarcoma-associated herpesvirus infection |
| 13 | 2.1215e-06 | Cellular senescence |
| 14 | 3.926e-06 | Prostate cancer |
| 15 | 4.7085e-06 | Human papillomavirus infection |
| 16 | 6.9853e-06 | Renal cell carcinoma |
| 17 | 7.9690e-06 | Focal adhesion |
| 18 | 1.1996e-05 | Endometrial cancer |
| 19 | 1.9017e-05 | Pancreatic cancer |
| 20 | 2.2339e-05 | Fc gamma R-mediated phagocytosis |
| 21 | 3.1626e-05 | Thermogenesis |
| 22 | 3.8218e-05 | Insulin signaling pathway |
| 23 | 4.2349e-05 | HIF-1 signaling pathway |
| 24 | 5.7459e-05 | Cell cycle |
| 25 | 5.5573e-05 | Regulation of actin cytoskeleton |
| 26 | 7.3416e-05 | Small cell lung cancer |
| 27 | 9.0859e-05 | Glioma |
| 28 | 0.00010078 | Circadian rhythm |
| 29 | 0.00010707 | FoxO signaling pathway |
| 30 | 0.00016942 | Hedgehog signaling pathway |

**Table S5. The top-30 pathways derived from joint lists of dysregulated mRNAs, miRNAs and lcnRNAs.**

|  | p-value | pathway |
| --- | --- | --- |
| 1 | 5.3117e-13 | Cell cycle |
| 2 | 2.8925e-10 | mTOR signaling pathway |
| 3 | 7.0109e-10 | Adherens junction |
| 4 | 6.6159e-09 | Cellular senescence |
| 5 | 1.3124e-08 | Prostate cancer |
| 6 | 2.1295e-08 | AMPK signaling pathway |
| 7 | 6.4517e-08 | Hippo signaling pathway |
| 8 | 7.5999e-08 | Chronic myeloid leukemia |
| 9 | 7.5999e-08 | Pancreatic cancer |
| 10 | 1.4628e-07 | Focal adhesion |
| 11 | 2.4929e-07 | Neurotrophin signaling pathway |
| 12 | 2.6210e-07 | Regulation of actin cytoskeleton |
| 13 | 5.3729e-07 | Hepatocellular carcinoma |
| 14 | 5.8077e-07 | EGFR tyrosine kinase inhibitor resistance |
| 15 | 8.9398e-07 | Kaposi sarcoma-associated herpesvirus infection |
| 16 | 2.3457e-06 | Human papillomavirus infection |
| 17 | 2.7670e-06 | Insulin signaling pathway |
| 18 | 5.3659e-06 | Thermogenesis |
| 19 | 6.9649e-06 | Longevity regulating pathway |
| 20 | 9.3850e-06 | Sphingolipid signaling pathway |
| 21 | 9.3569e-06 | FoxO signaling pathway |
| 22 | 9.3803e-06 | Thyroid hormone signaling pathway |
| 23 | 8.9597e-06 | Insulin resistance |
| 24 | 8.9597e-06 | HIF-1 signaling pathway |
| 25 | 1.4888e-05 | MAPK signaling pathway |
| 26 | 1.9862e-05 | Endocrine resistance |
| 27 | 3.1767e-05 | T cell receptor signaling pathway |
| 28 | 3.7747e-05 | Glioma |
| 29 | 4.1694e-05 | Renal cell carcinoma |
| 30 | 4.8095e-05 | Fc gamma R-mediated phagocytosis |
